# Bub2 regulation of cytokinesis and septation in budding yeast

**DOI:** 10.1186/1471-2121-10-43

**Published:** 2009-06-02

**Authors:** Su Young Park, Addie E Cable, Jessica Blair, Katherine E Stockstill, Katie B Shannnon

**Affiliations:** 1 Department of Biological Sciences, Missouri University of Science and Technology, Rolla, MO USA

## Abstract

**Background:**

The mitotic exit network (MEN) is required for events at the end of mitosis such as degradation of mitotic cyclins and cytokinesis. Bub2 and its binding partner Bfa1 act as a GTPase activating protein (GAP) to negatively regulate the MEN GTPase Tem1. The Bub2/Bfa1 checkpoint pathway is required to delay the cell cycle in response to mispositioned spindles. In addition to its role in mitotic exit, Tem1 is required for actomyosin ring contraction.

**Results:**

To test the hypothesis that the Bub2 pathway prevents premature actin ring assembly, we compared the timing of actin ring formation in wild type, *bub2Δ*, *mad2Δ*, and *bub2Δmad2Δ *cells both with and without microtubules. There was no difference in the timing of actin ring formation between wild type and mutant cells in a synchronized cell cycle. In the presence of nocodazole, both *bub2Δ *and *mad2Δ *cells formed rings after a delay of the same duration. Double mutant *bub2Δmad2Δ *and *bfa1Δmad2Δ *cells formed rings at the same time with and without nocodazole. To determine if Bub2 has an effect on actomyosin ring contraction through its regulation of Tem1, we used live cell imaging of Myo1-GFP in a *bub2Δ *strain. We found a significant decrease in the total time of contraction and an increase in rate of contraction compared to wild type cells. We also examined myosin contraction using Myo1-GFP in cells overexpressing an epitope tagged Bub2. Surprisingly, overexpression of Bub2 also led to a significant increase in the rate of contraction, as well as morphological defects. The chained cell phenotype caused by Bub2 overexpression could be rescued by co-overexpression of Tem1, and was not rescued by deletion of *BFA1*.

**Conclusion:**

Our data indicate that the Bub2 checkpoint pathway does not have a specific role in delaying actin ring formation. The observed increase in the rate of myosin contraction in the *bub2Δ *strain provides evidence that the MEN regulates actomyosin ring contraction. Our data suggest that the overexpression of the Bub2 fusion protein acts as a dominant negative, leading to septation defects by a mechanism that is Tem1-dependent.

## Background

The coordination of multiple events during M phase is essential for accurate chromosome segregation. Mitotic checkpoints ensure that chromosomes and the spindle are correctly aligned, and mitotic kinase activity prevents cytokinesis from occurring before mitosis is completed. Budding yeast is an excellent model system to study the complex regulation that couples mitotic exit with the onset of actomyosin ring contraction to complete cell division.

The mitotic checkpoint in budding yeast has two separate pathways, one for sensing kinetochore attachment to microtubules, and another for sensing spindle position [1]. The first pathway requires a number of known genes, including *MAD2*, and acts to prevent the metaphase to anaphase transition [2]. The second pathway relies on the Bub2/Bfa1 complex to inhibit mitotic exit [3-6]. Both pathways must be intact for complete arrest of the cell cycle in mitosis in response to microtubule depolymerization, as deletion of genes in a single pathway results in a cell cycle delay after which cells exit mitosis [3-6].

The spindle orientation pathway inhibits exit from mitosis through negative regulation of the mitotic exit network (MEN) [4]. Tem1, a small GTPase at the top of the pathway, is activated by its GEF Lte1, and inactivated by the two part GAP Bub2/Bfa1 [7-9]. The MEN regulates both mitotic exit and the onset of cytokinesis and septation. MEN activation leads to mitotic cyclin degradation, and the inactivation of mitotic kinase activity is required for cytokinesis [10,11]. In addition, some MEN proteins localize to the bud neck at the time of cytokinesis, and Tem1 has been shown to be required for myosin contraction [12-16]. Examination of actin ring formation in *apc2–8 *mutants with and without a *BUB2 *deletion led to the hypothesis that the Bub2 pathway acts to prevent premature actin ring formation [17,18]. This model predicts that *bub2Δ *cells will form actin rings earlier than *BUB2 *cells under conditions where the Bub2 checkpoint pathway is normally activated. It has also been proposed that Bub2 acts every cell cycle to restrain Tem1 activity until the end of mitosis [18], in which case even unperturbed cells lacking Bub2 may show premature actin ring formation. Here, we test these hypotheses by examining actin ring formation in synchronized cells with and without nocodazole to depolymerize microtubules and cause checkpoint activation. We found no evidence that the Bub2 checkpoint pathway acts to prevent premature actin ring formation, but we did see an effect on myosin contraction in *bub2Δ *cells, as well as a septation defect in cells overexpressing a dominant negative Bub2.

## Results

### The Bub2 checkpoint pathway does not regulate actin ring formation

**Figure 1:**
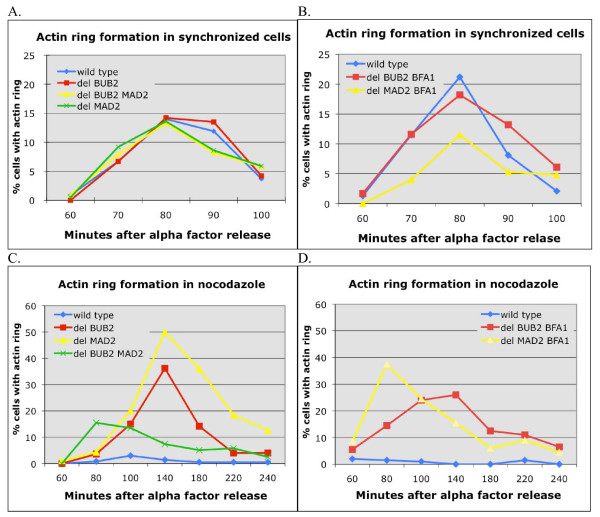
**The Bub2 checkpoint pathway does not regulate actin ring formation**. A. and B. Actin ring formation in synchronized cells. Isogenic wild type (KSY3), *bub2Δ *(KSY19), *bub2Δmad2Δ *(KSY47), *mad2Δ *(KSY51), *bub2Δbfa1Δ *(KSY110) and *mad2Δbfa1Δ *(KSY111) strains were arrested in G1 with α mating factor, released from arrest and fixed at regular intervals during mitosis. Cells were stained for actin with Alexa 586 phalloidin and ≥ 300 cells were examined microscopically at each time point to calculate the percentage of cells with an actin ring. Reproducible results were obtained in three independent experiments, and representative results from a single experiment are shown. C. and D. Actin ring formation in nocodazole. The same strains as in A. and B. were treated as above, except for the addition of nocodazole at release from α mating factor (time 0).

Previous studies showed that deletion of *BUB2 *led to MEN dependent actin ring formation in an *apc2–8 *mutant at the non-permissive temperature [17,18]. This led to the hypothesis that the Bub2 branch of the spindle assembly checkpoint specifically prevents premature actin ring formation. To test this idea, we looked at the timing of actin ring assembly in synchronized cells. Wild-type, *bub2Δ*, *mad2Δ*, and *bub2Δmad2Δ *cells were arrested in G1 using α-mating factor. After release from the arrest, cells were fixed at 10-minute intervals as they progressed through the cell cycle and stained for actin filaments. As shown in Figure 1A, actin ring formation peaked 80 minutes after release from G1 arrest in all strains. Therefore, deletion of *BUB2 *did not noticeably affect the timing of actin ring formation in a normal cell cycle.

We next examined actin ring formation after perturbation of the cell cycle using nocodazole, a drug that causes microtubule disassembly. As before, wild-type, *bub2Δ*, *mad2Δ*, and *bub2Δmad2Δ *cells were arrested in G1 using α-mating factor. Cells were released from the arrest into media containing 15 μg/ml nocodazole, fixed at 10-minute intervals, and stained for actin filaments. As expected, wild type cells did not form an actin ring due to cell cycle arrest (Figure 1C). Cells lacking either *BUB2 *or *MAD2 *formed actin rings with the same kinetics, with actin rings peaking at 140 minutes after release from G1 arrest (Figure 1C). The delay in actin ring formation in these strains relative to untreated cells is consistent with the cell cycle delay caused by loss of one of the two spindle damage checkpoints [3-6]. Double mutant *bub2Δmad2Δ *cells had peak actin ring formation at 80 minutes (Figure 1C), the same as unperturbed cells (Figure 1A), consistent with the complete loss of mitotic spindle checkpoints in the double mutant.

Our data show that actin ring formation in the presence of nocodazole occurs at the same time in *bub2Δ *cells as *mad2Δ *cells. Additionally, *bub2Δmad2Δ *cells form rings at the same time with or without microtubules. Therefore, Bub2 does not specifically restrain actin ring formation in response to spindle damage.

### Bfa1 does not show a Bub2 independent effect on actin ring formation

It has been proposed that Bfa1 has Bub2 independent functions, as overexpression of Bfa1 causes mitotic arrest even in the absence of Bub2 [6,19]. Therefore, we analyzed actin ring formation in synchronized *bub2Δbfa1Δ *and *mad2Δbfa1Δ *cells with and without nocodazole to see if deletion of *BFA1 *had an additional effect. In unperturbed cells, actin ring formation peaked at 80 minutes in wild type controls as well as *bub2Δbfa1Δ *and *mad2Δbfa1Δ *cells, the same as in *bub2Δ *and *mad2Δ *cells (Figure 1A and 1B). In nocodazole, *bub2Δbfa1Δ *cells had a peak of actin ring formation at 140 minutes, the same as in *bub2Δ *cells (Figure 1C and 1D). Nocodazole treated *mad2Δbfa1Δ *cells had a peak of actin ring formation at 80 minutes, similar to *mad2Δbub2Δ *cells in nocodazole and untreated cells (Figure 1C and 1D). Therefore, we found no evidence for Bfa1 having a Bub2 independent effect on actin ring formation.

### The rate of myosin contraction is affected by Bub2

**Figure 2:**
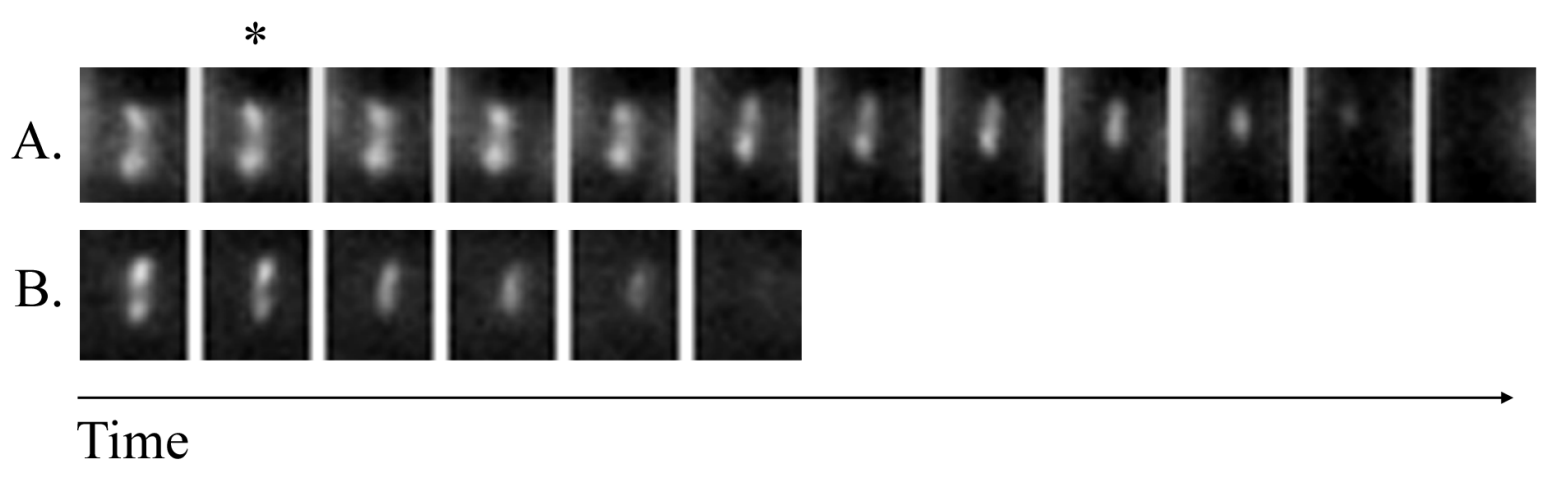
**Deletion of Bub2 decreases the time of myosin contraction**. A. Wild type cells expressing Myo1-GFP (KSY70) show contraction of myosin over 10 minutes. B. *bub2Δ *cells expressing Myo1-GFP (KSY71) show contraction of myosin over 4 minutes. The asterisk indicates the frame (T = 0) after which contraction is evident. Images in the series were taken at one-minute intervals.

Previous studies have suggested that the MEN regulates actomyosin ring contraction [14]. To test this hypothesis, we examined myosin contraction using Myo1-GFP in *bub2Δ *cells and compared myosin dynamics in *bub2Δ *cells to wild type and *mad2Δ*. Cells expressing Myo1-GFP were filmed at one-minute intervals on agarose pads and analyzed as described in Methods. Cells had a band of Myo1-GFP fluorescence across the bud neck that began to decrease in size at the onset of cytokinesis (Figure 2). The total time of contraction was measured from the time when the Myo1-GFP band began to decrease in size until Myo1-GFP was no longer visible at the bud neck (Figure 2 and Table 1). For wild type cells, the time of contraction was an average of 8.1 min (+/- 0.9), consistent with previously published results (Table 1, Figure 2A, and Additional File 1) [20,21]. Deletion of *BUB2 *resulted in a more rapid contraction, 5.9 min (+/- 1.3), (P < .005, Table 1, Figure 2B, and Additional File 2). In contrast, deletion of *MAD2 *resulted in a slightly slower average time of Myo1-GFP contraction, 10.1 min (+/- 3.5), although this difference was not significantly different from wild type (Table 1). We also calculated the average rate of contraction by measuring the width of the bud neck and dividing by the time of contraction. Deletion of *BUB2 *resulted in a significantly increased rate, 0.23 μm/min, compared to 0.16 μm/min in wild type cells (Table 1). The rapid contraction of the Myo1-GFP ring in *bub2Δ *cells prevented the observation of a clear "dot" of myosin signal normally seen at the bud neck at the end of cytokinesis (Figure 2). Analysis of fluorescence intensity profiles indicated that the Myo1-GFP signal ingressed equally from both sides without a loss of intensity. Overall, this data shows that the Bub2 checkpoint pathway has an effect on the timing of actomyosin ring contraction, while the Mad2 branch of the pathway does not.

**Table 1:** Deletion and overexpression of Bub2 increase the rate of myosin contraction.

Strain	Average size bud neck in μm +/- stdev	Average time of contraction in min +/- stdev	P value	Average rate of contraction μm/min +/- stdev	P value	N
wild type KSY70	1.3 +/- 0.2	8.1 +/- 0.9		0.16 +/- .04		7

bub2Δ KSY71	1.3 +/- 0.2	5.9 +/- 1.3	0.004	0.23 +/- .05	0.01	7

mad2Δ KSY73	1.3 +/- .06	10.1 +/- 3.5	0.3	0.14 +/- .08	0.5	4

Bub2 OE KSY132	1.3 +/- .09	6.3 +/- 0.5	0.001	0.21 +/- .02	0.03	7

### Bub2 overexpression affects myosin contraction and causes septation defects

Since *bub2Δ *caused an increase in the rate of contraction, we wanted to determine if overexpression of Bub2 would slow contraction. Bub2 tagged at the N terminus with T7 and at the C terminus with 6His under control of the inducible *GAL1 *promoter [19] was integrated into wild type cells expressing Myo1-GFP. These cells were grown in YPR, and then arrested in G1 with α factor with the addition of galactose for three hours to induce Bub2 expression. After release from G1 arrest, Myo1-GFP images were captured as cells underwent cytokinesis. Unexpectedly, Bub2 overexpression increased the speed and rate of Myo1 contraction similar to *bub2Δ *(Table 1). This result could be due to the overexpressed Bub2, which is tagged with T7 and 6His, acting as a dominant negative. See Additional file 3 for a time-lapse image of Myo1-GFP following Bub2 overexpression.

**Figure 3:**
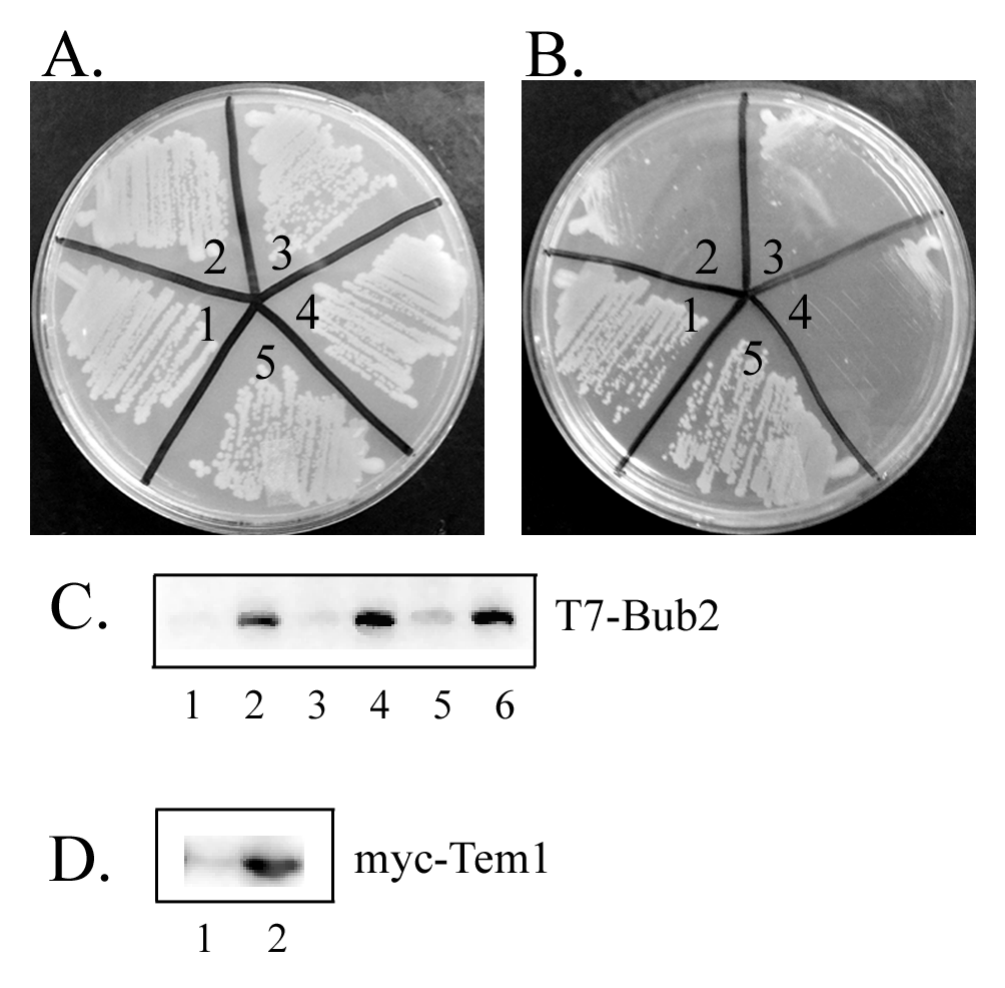
**Overexpression of a Bub2 construct slows growth**. A. Cells grown on YPD at 30°C for two days. B. Cells grown on YPGR at 30°C for two days. 1.) wild type (KSY3), 2.) wild type with GAL1-BUB2 (KSY129), 3.) *bub2Δ *with GAL1-BUB2 (KSY130), 4.) *bfa1Δ *with GAL1-BUB2 (KSY169) 5.) wild type with GAL1-Tem1 and GAL1-Bub2 (KSY171). C. GAL1-T7-Bub2-6His is expressed in galactose and repressed in glucose. Anti-T7 blot of protein extracts lane 1: wild type with GAL1-BUB2 (KSY129) grown in YPD, lane 2: wild type with GAL1-BUB2 (KSY129) grown in YPGR, lane 3: *bub2Δ *with GAL1-BUB2 (KSY130) grown in YPD, lane 4: *bub2Δ *with GAL1-BUB2 (KSY130) grown in YPGR, lane 5: *bfa1Δ *with GAL1-BUB2 (KSY169) grown in YPD, lane 6: *bfa1Δ *with GAL1-BUB2 (KSY169) grown in YPGR. D. Expression of GAL1-TEM induced by galactose and repressed by glucose. Anti-myc blot of protein extracts lane 1: GAL1-Tem1 GAL1-Bub2 (KSY171) in YPD, lane 2: GAL1-Tem1 GAL1-Bub2 (KSY171) in YPGR.

We further investigated the effects of T7-Bub2-6His overexpression by comparing cells with the GAL1-T7-Bub2-6His construct grown in conditions to repress (YPD) or induce (YPGR) expression. All strains showed a similar level of Bub2 protein expression when grown in YPGR (Figure 3C). As shown previously, cells exhibited slower growth on YPGR plates compared to YPD [19]. The slow growth was evident even in *bub2Δ *and *bfa1Δ *backgrounds (Figure 3A and 3B). This growth defect was rescued by co-overexpression of Tem1 using the *GAL1 *promoter (Figure 3A, B, and 3D). These data support the idea that the T7-Bub2-6His acts as a dominant negative.

**Figure 4:**
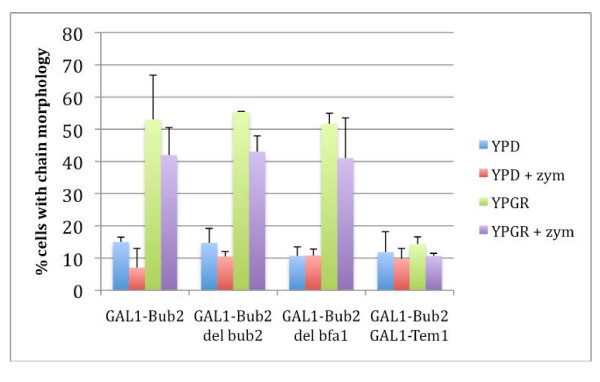
**Bub2 overexpression causes cytokinesis and septation defects that are rescued by co-overexpression of Tem1**. Cells were grown overnight (15 hours) in YPD or YPGR, then fixed. Strains used were: GAL1-BUB2 (KSY 129), GAL1-BUB2 *bub2Δ *(KSY130), GAL1-BUB2 *bfa1Δ *(KSY169), and GAL1-Bub2 GAL1-Tem1 (KSY171). Cells with (+ zym) and without zymolase treatment were examined under a microscope and the percentage of cells with chains (3 or more cell bodies) was calculated. Results shown are an average of three independent experiments, and error bars represent the standard deviation. 200 cells were counted for each treatment.

The overexpression of this Bub2 construct has been reported to cause a cytokinesis defect, although the data was not shown [19]. To study this effect more closely, we determined effects on cell morphology by examining cells grown in YPD or YPGR with and without zymolase treatment to remove the cell wall. In a wild type background, cells with the *GAL1*-T7-Bub2-6His construct grown on YPD had a very low incidence of the chain phenotype, defined as three or more cell bodies (Figure 4). After induction of Bub2 overexpression, an average of 53% of cells exhibited the chained phenotype (Figure 4). After treatment with zymolase, the chains were reduced to 42% on average (Figure 4). In support of the idea that *GAL1*-T7-Bub2-6His acts in a dominant negative fashion, the effect of overexpression was similar in a bub2Δ strain, with 55.5% of bub2Δ cells exhibiting a chain phenotype before zymolase, and 43% after zymolase treatment (Figure 4). If the epitope-tagged Bub2 were acting the same as endogenous Bub2, one would expect the phenotype to be relieved, rather than exacerbated, by deletion of *BUB2*. This data also shows that deletion of *BUB2 *does not cause a cytokinesis or septation defect, as wild type cells and *bub2Δ *cells had similar numbers of chained cells on YPD (Figure 4). Unlike the previous report [19], we did not find that deletion of *BFA1 *rescued the chain phenotype (Figure 4). The morphological defects were rescued by co-overexpression of Tem1, which reduced the percentage of chained cells in YPGR to similar levels as in YPD (Figure 4). Together, our data shows that overexpression of a T7-Bub2-6His fusion protein acts as a dominant negative, causing a chained cell phenotype that can be rescued by Tem1 overexpression. The results of the zymolase assay suggest that overexpression of Bub2 caused both a cytokinesis and a septation defect.

**Figure 5:**
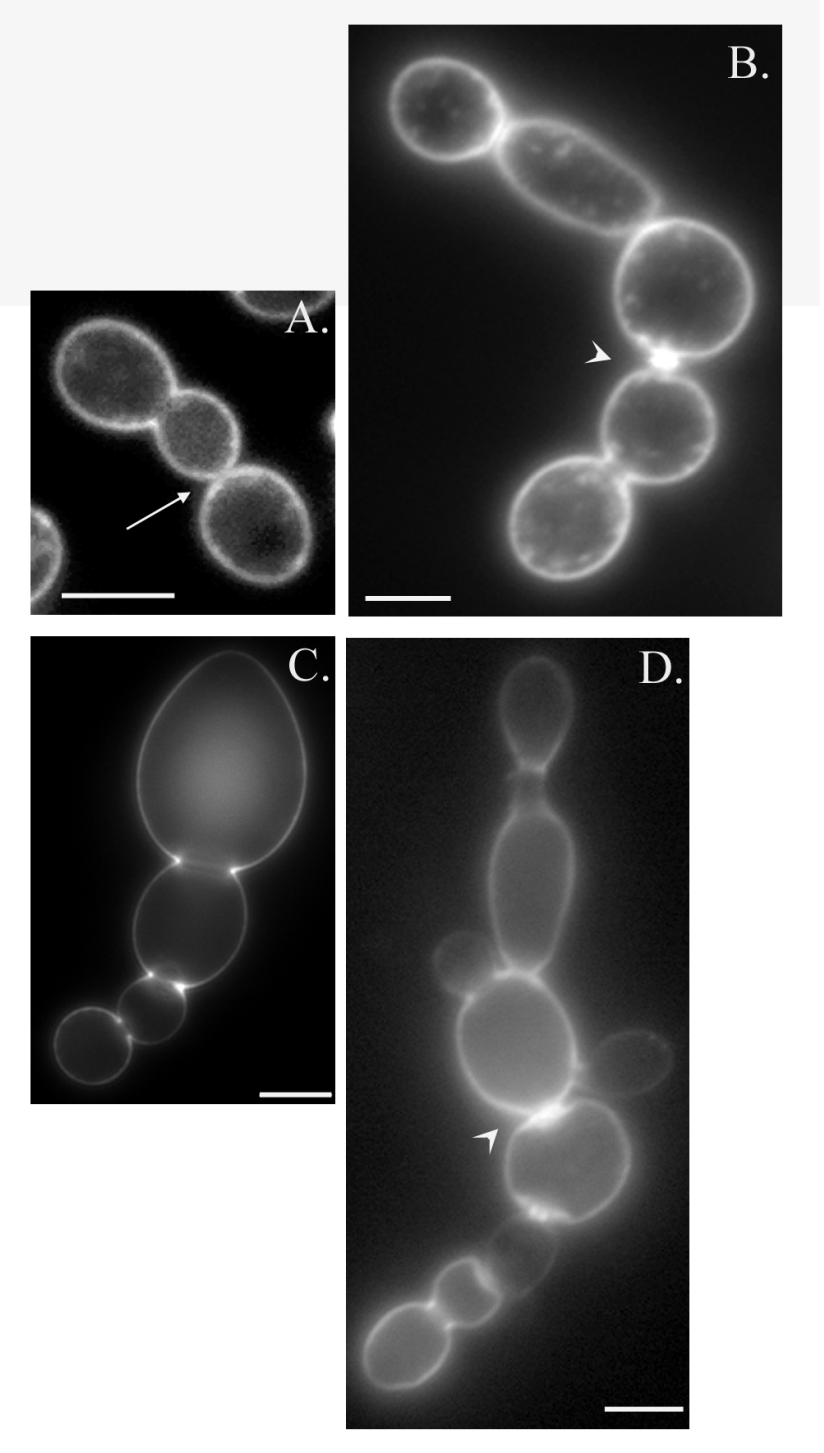
**Bub2 overexpression causes a defect in cell wall formation**. Wild type cells with GAL1-BUB2 (KSY 129) were grown in YPGR for eight (A. and C.) or fifteen (B. and C.) hours. A. and B. FM4-64 staining to visualize the plasma membrane. Arrow points to closed bud necks. Arrowhead points to abnormal plasma membrane at the bud neck. C. Calcofluor staining shows lack of septum between buds in chains. D. Calcofluor staining shows abnormal septum formation after longer incubation in YPGR (arrowhead). Images are a single plane from a Z series and represent open or closed bud necks as determined from examination of all planes of the series. Scale bar = 5 μm.

Because the percentage of chains caused by Bub2 overexpression decreased about 10% in zymolase, we examined plasma membrane and cell wall formation after Bub2 overexpression. The plasma membrane was visualized using FM 4–64, and the cell wall was stained using Calcofluor. Examination of chains with three of four cell bodies, formed after eight hours of Bub2 overexpression, showed that chains typically had plasma membrane across some bud necks, but not others (Figure 5A). In contrast, these chains showed a complete lack of cell wall formation at all bud necks (Figure 5C). This result was surprising, since the zymolase assay (Figure 4) suggested greater defects in cytokinesis than septation. Therefore, we repeated the analysis after fifteen hours in YPGR. Most bud necks showed plasma membrane formation between cell bodies, although in some cases the plasma membrane had abnormal bright dots or clumps at the bud neck (Figure 5B). Only the buds at the end of the chains did not have plasma membrane, consistent with being the most recently formed buds. Cell wall formation looked very different after the longer Bub2 induction, with most bud necks now showing septum formation, although in many cases the septa seemed thicker and brighter than normal (Figure 5D). These results suggest that overexpression of the dominant negative Bub2 causes primary septum formation to fail, but over time cells may be able to separate by forming an abnormal secondary septum (see Discussion).

## Discussion

### Regulation of actin ring formation

The MEN pathway in budding yeast regulates cytokinesis as well as exit from mitosis. However, how the MEN controls cytokinesis is not understood. Experimental evidence that actin rings form in *apc2–8 bub2Δ *cells suggested that the Bub2 checkpoint pathway prevents premature actin ring formation [17,18]. However, this hypothesis is inconsistent with data showing that Tem1, the target of Bub2 regulation, is not required for actin ring formation [14].

Our examination of actin ring formation in *bub2Δ *and *bfa1Δ *strains shows that actin ring formation occurs with normal kinetics in the absence of the Bub2 checkpoint. Although it is possible that Bub2 or Bfa1 has a subtle effect on actin ring formation that was missed by the 10 minute intervals used to examine the cells, this seems unlikely, as our data is consistent with previous results showing that *bub2Δ *does not advance anaphase onset [5].

Bub2 and Mad2 are required for two separate branches of the mitotic spindle checkpoint in budding yeast. Our data show that cells lacking either one of these checkpoint pathways form actin rings with the same kinetics after nocodazole treatment. Therefore, the delay in actin ring formation in *bud2Δ *and *mad2Δ *cells is caused by a cell cycle delay. The actin rings seen in *apc2–8 bub2Δ *cells [17,18] are likely due to loss of cell cycle arrest and subsequent downstream events such as actin ring formation. In fact, the authors noted that *apc2–8 *cells remained arrested in mitosis, while *apc2–8 bub2Δ *cells exited mitosis after two hours [18]. Therefore, progression through late anaphase, when MEN mutants arrest, is required for actin ring formation, but there is no evidence that the MEN regulates actin ring formation directly.

Our data also do not support a Bub2 independent function of Bfa1 in actin ring formation. There was no apparent difference in the timing of actin ring formation in unperturbed or nocodazole treated cells in isogenic strains with and without a *BFA1 *deletion.

### The MEN and actomyosin ring contraction

One way the MEN may act to regulate cytokinesis is to trigger the onset of myosin contraction. Our data show that deletion of *BUB2 *or overexpression of a dominant negative Bub2 decreases the total time for contraction of the myosin ring and increases the rate of contraction. This effect on myosin contraction could either be due to direct regulation of contraction by the MEN or an indirect effect caused by defects in primary septum formation. Previous studies have shown that preventing primary septum synthesis results in a cytokinesis failure due to premature breaking and disassembly of the actomyosin ring [22,23].

We do not think our results are due to indirect effects of septation defects for two reasons. Firstly, we did not see any evidence of abnormal or asymmetrical contraction in *bub2Δ *or *GAL1*-T7-Bub2-6His cells, which would be predicted by this model. Secondly, *bub2Δ *cells showed no evidence of a defect in primary septum formation, yet still exhibited the myosin contraction phenotype. Our results suggest that MEN activation directly influences either the onset or the rate of myosin contraction, and that the Bub2 checkpoint pathway acts as a brake on actomyosin ring contraction. This model is also supported by previous results showing that Tem1 and Mob1 are required for actomyosin ring contraction when the requirement for MEN function in mitotic exit is bypassed [14,24]. The MEN therefore has a role in promoting cytokinesis that is separate from its role in mitotic kinase inactivation. In contrast, the role of the MEN in promoting primary septum formation depends only on exit from mitosis [25]. The identification of MEN targets involved in the regulation of actomyosin ring contraction will be of great interest. Potential targets include Iqg1, which has been shown to interact with Tem1 *in vitro*, and Hof1, which is phosphorylated in a MEN-dependent manner [26,27].

### Dominant negative effects of Bub2 overexpression

The *GAL1*-T7-Bub2-6His construct used in this study has been previously described as partially inhibiting cell growth only in the presence of Bfa1 [19]. It was also reported to cause a chained cell phenotype, although this data was not shown. We have confirmed that this construct causes a mild growth defect as well as a chain phenotype on galactose-containing media in our strain background. However, we did not find that deletion of *BFA1 *rescued either the growth defect or the chain morphology caused by overexpression of the Bub2 fusion protein. We have further examined the chained cell phenotype, and found evidence for the presence of both cytokinesis and septation defects.

We obtained this construct in the hopes that overexpressing Bub2 would decrease the rate of myosin contraction, since deletion of Bub2 increased the rate. However, we found similar rates of Myo1-GFP contraction in cells expressing T7-Bub2-6His and *bub2Δ *cells. This is most likely due to the T7-Bub2-6His acting as a dominant negative that interferes with normal Tem1 function. Supporting this conclusion, overexpression of an untagged Bub2 using the *GAL1 *promoter does not result in a growth defect [28]. Interestingly, overexpression of Bfa1 has a different phenotype than overexpression of either untagged or dominant negative Bub2. Bfa1 overexpression arrests cells in mitosis, and this arrest does not require Bub2 [6,19].

### Causes of cytokinesis and septation defects

Interestingly, we did not observe a correlation between increased rates of myosin contraction and cytokinesis or septation defects. Deletion of *BUB2 *or overexpression of Bub2 had a similar effect on Myo1-GFP dynamics, but only Bub2 overexpression resulted in the chained cell phenotype. Therefore, the significantly increased rate of myosin contraction we observed does not have an effect on cell morphology under normal growth conditions.

How then does overexpression of a dominant negative Bub2 cause morphological defects? Our data suggest that the T7-Bub2-6His must be affecting Tem1 in a way that does not prevent exit from mitosis, but perturbs septum formation. A temperature sensitive mutant of the MEN kinase Cdc15, *cdc15-2*, has been shown to have a septation defect when grown at the semipermissive temperature (31°C) [13]. Since binding of Cdc15 to Tem1 is required for Cdc15 activation and prevented by Bub2/Bfa1, the interaction of Cdc15 with Tem1 could be partly inhibited by T7-Bub2-6His overexpression [29,30].

Zymolase treatment of cells after fifteen hours of Bub2 overexpression resulted in approximately 10% fewer chains than without zymolase, suggesting that some of the chains were due to septation failure, and most of the chains were due to cytokinesis failure. However, staining of plasma membrane and cell wall structures at earlier timepoints showed that lack of septum formation was the primary defect (Figure 5C). Staining at later timepoints indicated that many of the bud necks in the chains had become separated by plasma membrane and chitin, some of which appeared abnormal (Figure 5) and may represent the deposition of thick secondary septa that eventually separate the cells, sometimes trapping pockets of membrane within. Electron microscopy has shown that yeast cells that fail cytokinesis due to lack of myosin and those that fail to form the primary septum due to a lack of chitin synthase have identical phenotypes [23]. In both cases, the cells form a thick cell wall that lack the normal trilaminar structure [23]. It is unclear whether or not zymolase can efficiently digest this abnormally thick septum. Therefore, the zymolase assay may not be able to distinguish failure to contract an actomyosin ring from failure to build a primary septum, since in both cases the cells can eventually separate the plasma membrane by lateral thickening of the cell wall. Additionally, it has been shown that preventing primary septum formation causes instability of the actomyosin ring [22]. Therefore, the two processes of cytokinesis and septation may be so closely linked in budding yeast that defects in one process result in problems in the other.

We think it is likely that the formation of chains of cells after overexpression of a dominant negative Bub2 is caused by a failure to form the primary septum, and that these cells eventually form an abnormal septum as seen previously by EM. The contradictory results from the zymolase assay may be due to incomplete digestion of these thick septal structures, failing to separate the chained cell bodies even though they are separated by plasma membrane. Together, our data suggest that the Bub2 pathway, and therefore the MEN, regulates septation or the coordination of septation with actomyosin ring contraction.

## Conclusion

Deletion of *BUB2 *did not affect the timing of actin ring assembly, but did increase the rate of myosin contraction. Overexpression of a tagged Bub2 protein caused septation defects that were rescued by Tem1 overexpression. Together, the data suggest that the MEN regulates actomyosin ring contraction and septation.

## Methods

### Yeast media

Yeast cell culture and genetic techniques were carried out by methods described [31]. Yeast cells were grown in YPD medium (1% yeast extract, 2% Bacto peptone, 2% glucose), YPR (1% yeast extract, 2% Bacto peptone, 2% raffinose), or YPGR (1% yeast extract, 2% Bacto peptone, 2% galactose, 2% raffinose) liquid medium, or on – HIS, -LEU, -TRP or -URA drop-out solid and/or liquid medium at 30°C.

### Strain Construction

All *S. cerevisiae *strains were W303 background, derived from KSY3 (*MATa ura3-52 leu2-3,112 his3-Δ200 trp1-1 ade2 Δbar1*). All yeast strains used for this study are listed in Table 2. To create Myo1-GFP expressing strains, pKT36 (MYO1 under its endogenous promoter tagged with GFP on a TRP1 CEN plasmid) [14] was digested with *AgeI *enzyme and transformed into KSY3, KSY19, KSY51, and KSY129 strains to make KSY 70, 71, 73, and 132 respectively.

**Table 2:** Strains used in this study. All strains are W303 background.

Strain Name	Genotype	Source
KSY3	*MATa ura3–52 leu2–3,112 his3-Δ200 trp1-1 ade2 Δbar1*	Lippincott and Li, 1998 (RLY261)

KSY19	*MATa ura3–52 leu2–3,112 his3-Δ200 trp1-1 ade2 Δbub2:LEU2 Δbar1*	Li lab (RLY597)

KSY21	*MATa ura3–52 leu2–3,112 his3-Δ200 trp1-1 ade2 Δbfa1: HIS3 Δbub2:LEU2*	Li, 1999 (RLY684)

KSY22	*MATa ura3–52 leu2–3,112 his3-Δ200 trp1-1 ade2 Δbfa1: HIS3 Δmad2:LEU2*	Li, 1999 (RLY686)

KSY24	*MATa ura3–52 leu2–3,112 his3-Δ200 trp1-1 ade2 Δmad2:LEU2*	Li, 1999 (RLY718)

KSY25	*MATa ura3–52 leu2–3,112 his3-Δ200 trp1-1 ade2 Δbub2:LEU2 Δmad2:URA3*	Li, 1999 (RLY736)

KSY47	*MATa ura3–52 leu2–3,112 his3-Δ200 trp1-1 ade2 Δbub2:LEU2 Δmad2:URA3 Δbar1:HIS3*	This work

KSY51	*MATa ura3–52 leu2–3,112 his3-Δ200 trp1-1 ade2 Δmad2:LEU2 Δbar1:HIS3*	This work

KSY70	*MATa ura3–52 leu2–3,112 his3-Δ200 trp1-1 ade2 Δbar1 MYO1-GFP: TRP1 (pKT36)*	This work

KSY71	*MATa ura3–52 leu2–3,112 his3-Δ200 trp1-1 ade2 Δbub2:LEU2 Δbar1 MYO1-GFP: TRP1 (pKT36)*	This work

KSY73	*MATa ura3–52 leu2–3,112 his3-Δ200 trp1-1 ade2 Δmad2:LEU2 Δbar1:HIS3 MYO1-GFP: TRP1 (pKT36)*	This work

KSY110	*MATa ura3–52 leu2–3,112 his3-Δ200 trp1-1 ade2 Δbfa1: HIS3 Δbub2:LEU2 Δbar1:URA3*	This work

KSY111	*MATa ura3–52 leu2–3,112 his3-Δ200 trp1-1 ade2 Δbfa1: HIS3 Δmad2:LEU2 Δbar1:URA3*	This work

KSY129	*MATa ura3–52 leu2–3,112 his3-Δ200 trp1-1 ade2 Δbar1 GAL1-T7-BUB2-6His: URA3 (pKL1315)*	This work

KSY130	*MATa ura3–52 leu2–3,112 his3-Δ200 trp1-1 ade2 Δbub2:LEU2 Δbar1 GAL1-T7-BUB2-6His: URA3 (pKL1315)*	This work

KSY132	*MATa ura3–52 leu2–3,112 his3-Δ200 trp1-1 ade2 Δbar1 MYO1-GFP: TRP1 (pKT36) GAL1-T7-BUB2-6His: URA3 (pKL1315)*	This work

KSY167	*MATa ura3–52 leu2–3,112 his3-Δ200 trp1-1 ade2 Δbfa1: HIS3*	Li, 1999 (RLY683)

KSY169	*MATa ura3–52 leu2–3,112 his3-Δ200 trp1-1 ade2 Δbfa1: HIS3 GAL1-T7-BUB2-6His: URA3 (pKL1315)*	This work

KSY171	*MATa ura3–52 leu2–3,112 his3-Δ200 trp1-1 ade2 Δbar1 GAL1-T7-BUB2-6His: URA3 (pKL1315) GAL1-myc-Tem1 (pKT25)*	This work

To create Bub2 over-expressing strains, pKT1315 (Ro et al., 2001) was digested with *NcoI *and transformed into KSY3, KSY19 and KSY167 to make KSY129, 130, and 169 respectively. To create the GAL1-myc-TEM1 strain KSY171, pKT25 (GAL1-myc-TEM1 on a LEU2 CEN plasmid) was cut with XcmI and transformed into KSY129. Expression of Bub2 and Tem1 was verified by Western Blot.

To make strains more sensitive to alpha mating factor, the *BAR1 *locus was replaced by *HIS3 *or *URA3 *in strains KSY21, KSY22, KSY24, and KSY25 using PCR-mediated one-step gene replacement [32] using PRS313 or PRS316 as the template and primers as in [33] to make KSY110, KSY111, KSY51, and KSY47 respectively.

### Yeast Transformation

Yeast transformations were performed by modified lithium acetate method [34]. After 30 minutes incubation at 30°C, 50 μl of DMSO was added and mixed. Cells were plated on appropriate media and incubated at 30°C for two-three days.

### Fluorescence staining of Yeast Cells

KSY3, KSY19, KSY47, KSY51, KSY111 and KSY112 were grown in YPD overnight. α-factor was added to a final concentration of 100 ng/ml for 2 hours to synchronize cells. Cells were washed with sterile dH_2_O three times and resuspended in YPD or YPD with nocodazole (15 μg/ml final). After release, cells were incubated at 30°C. Cells released into YPD were incubated for 60 to 100 minutes, during this period at every ten minutes, 5 ml of cells were taken and fixed with 670 μl 37% formaldehyde for two hours at room temperature. Cells released in YPD with nocodazole were incubated for 60 to 240 minutes. At 60, 80, 100, 140, 180, 220 240 minutes time points, 5 ml of cells were collected and fixed as above. Fixed cells were stained with Alexa 568 (Invitogen) or rhodamine (Cytoskeleton Inc. Denver, CO) phalloidin as described [21].

Cells were observed using an Olympus IX51 inverted microscope at 1,000× total magnification using a UPLSAPO 100× NA 1.4 objective. A Texas-red filter (Brightline) was used to view phalloidin staining. Images were captured with a Hamamatsu ORCA285 CCD camera. Shutters, filters, and camera were controlled using Slide Book software (Intelligent Imaging Innovations, Denver, CO).

### Time-lapse microscopy

Cells were grown overnight and placed on agarose pads made by melting 0.2 g of agarose in 1 ml -TRP media [35]. Cells from strain KSY132 were grown overnight in YPR, then arrested with alpha factor with galactose added to 2% final for three hours. Cells were washed 3× with sterile water to release from arrest before imaging. Cells were placed on the agarose pad and covered with cover slip sealed with valap (1:1:1 mixture of Vaseline, lanolin, and paraffin). Living cells were viewed using an Olympus IX51 inverted microscope at 1,000× total magnification using a UPLSAPO 100× NA 1.4 objective. FITC (EX 482/35 506DM EM 536/40) filter was used to visualize GFP (Brightline). Images were captured with a Hamamatsu ORCA285 CCD camera. Shutters, filters, and camera were controlled using Slide Book software (Intelligent Imaging Innovations, Denver, CO). Images were collected with exposure (100 ms to 200 ms) to fluorescent light at 1 minute intervals.

### Analysis of myosin contraction

To determine the onset of myosin contraction, the time-lapse series of Myo1-GFP expressing cells was viewed at 200% magnification. Time zero was defined as the timepoint after which the ring decreased in diameter. The ruler tool in Slidebook was used to measure the ring at T = 0 and the following frame to confirm that a change in diameter had occurred. A second blind analysis to determine time zero was performed in order to eliminate observer bias. Total time of contraction was the time elapsed between time zero and when the Myo1-GFP signal disappeared from the bud neck. To determine the rate of contraction, the width of the bud neck was measured using the ruler tool in Slidebook and divided by the total time of contraction. Fluorescence intensity profiles were used to examine symmetry of ring contraction. Excel was used to calculate standard deviation and P-values using the two-tailed T test.

### Morphological Observations

Cultures of KSY129, 130, 169, and 171 were grown in YPR overnight, then diluted into YPD and YPGR and grown for fifteen hours at 30°C before addition of formaldehyde to 5% final. Fixed cells were treated both with and without zymolyase before observing cells under the microscope for phenotypic changes as described [21]. Morphology was observed and cells were counted under Olympus CH2, objective EA40 NA0.65.

### Immunoblot Analysis

Cultures of KSY129, 130, 169, and 171 were grown in YPR overnight, then diluted into either YPD or YPGR and grown for three hours at 30°C before total yeast extracts were prepared by glass bead lysis in SDS sample buffer. Extracts were run on 4–20% Tris-Glycine gels (Invitrogen) and transferred to nitrocellulose. T7-Bub2-6His was detected using anti-T7 antibody diluted 1:1000 (Novagen), and myc-Tem1 was detected using anti-myc monoclonal antibody diluted 1:1000 (Covance). Donkey anti-mouse HRP conjugated secondary antibody was diluted 1:2500 (Jackson ImmunoResearch). The Pierce chemiluminescence detection kit was used (Thermo Scientific). Comparable amounts of total protein were loaded in each lane as shown by Coomassie staining of the same extracts on a separate gel.

### Plasma membrane and cell wall staining

To visualize the plasma membrane, FM4–64 (Invitrogen) was added to cells in growth media on ice. For cell wall staining, Calcofluor was used as described [36]. Calcofluor (Fluorescent brightener 28, Sigma) was added to cells in growth medium to a final concentration of 100 μg/ml. Cells were incubated 5 minutes, washed twice with H_2_O, and observed with a DAPI filter set.

## Authors' contributions

SYP made strains, produced a majority of the data, and wrote Methods section. AC performed some of the live cell imaging experiments. JB made additional strains and produced data shown in Figure 4. KS participated in data analysis. KBS planned, supervised, and performed experiments and wrote the manuscript. All authors read and approved the final manuscript.

## Supplementary Material

Additional File 1 **Myo1-GFP in wild type cell**. This movie shows myosin contraction in the cell shown in Figure 2A. The movie begins at asterisk in the figure, and was taken at one-minute intervals. Click here for file

Additional File 2 **Myo1-GFP in a *bub2Δ *cell**. This movie shows myosin contraction in the cell shown in Figure 2B. The movie begins at asterisk in the figure, and was taken at one-minute intervals. Click here for file

Additional File 3 **Myo1-GFP in a cell overexpressing Bub2**. This movie shows myosin contraction in two cells after Bub2 induction by addition of galactose for three hours. The movie was taken at one-minute intervals. Click here for file
